# Diagnosis and Evaluation of Aggressiveness Using Circulating Plasma miRNAs in Papillary Thyroid Microcarcinoma

**DOI:** 10.3390/cancers17132079

**Published:** 2025-06-21

**Authors:** Jiwon Jang, Ji Min Kim, Sung-Chan Shin, Yong-il Cheon, Bo Hyun Kim, Mijin Kim, Sang Soo Kim, Byung-Joo Lee

**Affiliations:** 1 Department of Otorhinolaryngology-Head and Neck Surgery, College of Medicine, Pusan National University and Biomedical Research Institute, Pusan National University Hospital, Busan 49241, Republic of Korea; jjw07071@gmail.com (J.J.); ny5thav@hanmail.net (J.M.K.); cha-nwi@hanmail.net (S.-C.S.); skydragonone@naver.com (Y.-i.C.); 2 Department of Internal Medicine, College of Medicine, Pusan National University and Biomedical Research Institute, Pusan National University Hospital, Busan 49241, Republic of Korea; pons71@hanmail.net (B.H.K.); mijinkim08@gmail.com (M.K.); drsskim7@gmail.com (S.S.K.); 3 Department of Otorhinolaryngology-Head and Neck Surgery, Good-Gang-An Hospital, Busan 48265, Republic of Korea

**Keywords:** microRNA, papillary thyroid microcarcinoma, miRNA expression levels, aggressiveness

## Abstract

**Background/Objectives:** MicroRNAs are emerging as valuable diagnostic markers for various diseases, including papillary thyroid carcinoma (PTC). However, there is limited research on circulating miRNA expression in papillary thyroid microcarcinoma (PTMC). Therefore, we conducted a study to explore whether plasma-derived miRNAs can distinguish PTMC from benign nodules or predict aggressiveness. **Methods:** A total of 150 patients who underwent thyroidectomy from January 2013 to July 2021 were enrolled in this study. Patients were divided into three groups: benign, low-risk PTMC, and advanced PTMC. Nine patients from each group were selected for microarray analysis for plasma miRNAs. Six miRNAs were selected for comparison of expression levels using TaqMan assay. The ROC curve was utilized to evaluate the diagnostic and aggressiveness value of the miRNAs. **Results:** From the microarray analysis, miR-455-3p and miR-548ac were identified as miRNAs that can significantly differentiate between benign nodules and PTMC. A combination of six miRNAs (miR-455-3p, miR-548ac., miR-221, miR-222, miR-146a. miR-146b) rather than individual miRNAs had the highest AUC (0.857), sensitivity (0.867), and specificity (0.800) in differentiating benign and PTMC. In microarray analysis, no significant miRNAs were observed to distinguish between low-risk group and aggressive PTMC. However, in the six-miRNA combination, it was possible to distinguish low-risk PTMC from aggressive PTMC with an AUC of 0.763, sensitivity of 0.739, and the specificity of 0.727. **Conclusions:** A combination of six miRNAs presents the possibility of distinguishing between benign and PTMC and low-risk and aggressive PTMC with an acceptable AUC, sensitivity, and specificity.

## Simple Summary

This study investigated whether miRNAs can differentiate PTMCs (papillary thyroid microcarcinoma) from benign nodules and predict their aggressiveness. In a group of 150 thyroidectomy patients, a combination of six miRNAs (miR-455-3p, miR-548ac, miR-221, miR-222, miR-146a, and miR-146b-5p) demonstrated the best ability to distinguish between benign, low-risk, and advanced PTMCs. This miRNA combination was also effective in differentiating low-risk PTMCs from aggressive ones.

## 1. Introduction

Thyroid cancer is the most common malignancy of the endocrine system, with the incidence rate increasing since the early 1980s. It accounts about 3–4% of all human cancers [1]. Despite its prevalence, the 5-year relative survival rate remains high at 98.4%, which is among the highest across all cancer types [2,3]. Papillary thyroid carcinoma (PTC) is the most common type of thyroid cancer, accounting for approximately 84% of cases [2,4]. Advancements in diagnostic tools for thyroid cancer have led to increased detection at an early stage. Consequently, the incidence of papillary thyroid microcarcinoma (PTMC) has increased over the past 20 years across both sexes and among various races and ethnicities [5].

PTMCs are defined as PTC measuring less than 1cm in diameter. While high-resolution ultrasound has significantly improved the early detection of PTMCs, it has also raised concerns regarding overdiagnosis and subsequent overtreatment, particularly in clinically indolent cases [6]. In response, the concept of active surveillance (AS) has emerged as an alternative to immediate surgery for selected low-risk PTMCs without gross extrathyroidal extension (ETE) or clinical metastasis [7]. Current guidelines recommend periodic neck ultrasound initially every 6 months for the first year, and annually thereafter to monitor tumor progression in AS candidates.

For patients who are not candidates for AS, surgery is the standard treatment for PTMC. Lobectomy is generally recommended for unilobar PTMC with low risk on ultrasonography [5]. However, some cases like lateral neck metastases or other aggressive features require total thyroidectomy with lateral neck dissection followed by radioactive iodine (RAI) therapy. Since most aggressive characteristics are identified through postoperative histopathological examination, developing preoperative tools that can help distinguish patients with potentially advanced PTMC is needed.

Although fine needle aspiration (FNA) is commonly utilized to guide treatment decisions for thyroid nodules, its application in subcentimeter thyroid nodules remains technically challenging due to the small size. Medical professionals face frequent difficulties in obtaining adequate cytologic materials. In this regard, blood-based molecular diagnostic tools, which do not rely on tissue acquisition, could offer a promising non-invasive alternative. If a molecular tool could support the identification of candidates suitable for active surveillance and predict tumor aggressiveness, it could not only reduce unnecessary surgeries but also lower the frequency of reoperations after initial surgery.

Microribonucleic acids (miRNAs) are small ribonucleic acid (RNA) molecules, generally 20–24 nucleotides long, that serve as crucial regulators of gene expression at the post-transcriptional stage [8]. Alterations or malfunctions in miRNAs have been linked to different types of human cancers, suggesting that miRNAs play roles in inhibiting tumor growth and supporting oncogenes [9]. MiRNAs serve as significant biomarkers for predicting cancer progression in various types of malignancies [8,9,10]. The detection of miRNAs in the thyroid tissue was associated with the diagnosis of PTC, as well as their role in evaluating the aggressiveness of PTC, including lymph node metastasis [1,11,12,13]. The diagnosis and prognosis of thyroid cancer using different miRNAs, such as miR-221, miR-222, miR-146a, and miR-146b-5p, has been investigated in thyroid cancer tissues [14,15,16,17,18]. Although research on miRNAs for the diagnosis and prognosis of tissues after surgery is abundant, studies specifically focused on circulating miRNAs in PTMC remain limited. Owing to their stability in plasma, circulating miRNAs represent potential candidates for diagnostic and prognostic biomarkers in PTMC.

Therefore, we investigated whether these miRNAs maintain their diagnostic efficacy and predictive value for the prognosis of PTMC. We employed a two-step approach. First, we performed a microarray analysis to identify novel miRNAs present in the plasma that could differentiate between benign nodules and PTMCs and between low-risk and advanced PTMCs. Second, we evaluated whether previously reported tissue-based miRNAs, such as miR-221, miR-222, miR-146a and miR-146b, retain their diagnostic efficacy in plasma samples. This combined strategy was designed to explore novel circulating biomarkers while simultaneously validating known candidates. The goal of this study is to provide a non-invasive blood-based diagnosis tool to assist in deciding whether to pursue active surveillance and to determine the appropriate extent of surgical intervention in PTMC.

## 2. Patients and Methods

### 2.1. Ethics Approval

This research was performed with ethical approval from the Korean Human Bioresource Bank Network. All the patients who participated in the study provided informed consent. Blood samples were obtained from patients on the day of the operation and stored in the Busan National University Hospital Human Bioresource Bank. The Institutional Review Board reviewed and approved this study (H-2211-001-120).

### 2.2. Subjects

This study included patients diagnosed with benign nodules and PTMC after surgery at the Department of Otolaryngology, Pusan National University Hospital, from January 2013 to July 2021. Patients were included if an adequate number of stored preoperative plasma samples were available. Patients concurrently diagnosed with other types of malignancy were excluded to avoid confounding miRNA profiles and ensure specificity to PTMC. Ultimately, 150 patients were included in this study. A total of 150 patients and their characteristics are outlined in Table 1.

Patients were equally divided into three groups: benign nodules (*n* = 50), low-risk PTMC (*n* = 50), and advanced PTMC (*n* = 50). Sample size was determined using G Power 3.1.9.7 software (Heinrich Heine University, Dusseldorf, Germany), assuming a medium effect size (Cohen’s d = 0.5), a significance level of 0.05, and a power of 0.80. Our design met the minimum required sample size for all group comparisons. One hundred patients diagnosed with PTMC after surgery were selected and categorized into the low-risk and advanced PTMC groups. Frequency matching was used based on clinical characteristics such as age, sex, primary tumor size, and multifocality (Table 1).

Risk group classification was based on whether RAI therapy was recommended. Patients presenting with five or more central neck lymph node metastases, one or more lateral neck lymph node metastases, or gross extrathyroidal extension (ETE) were classified into the advanced PTMC group, according to the risk stratification system and considerations in RAI decision-making provided by the American Thyroid Association (ATA) [19].

**Figure 1:**
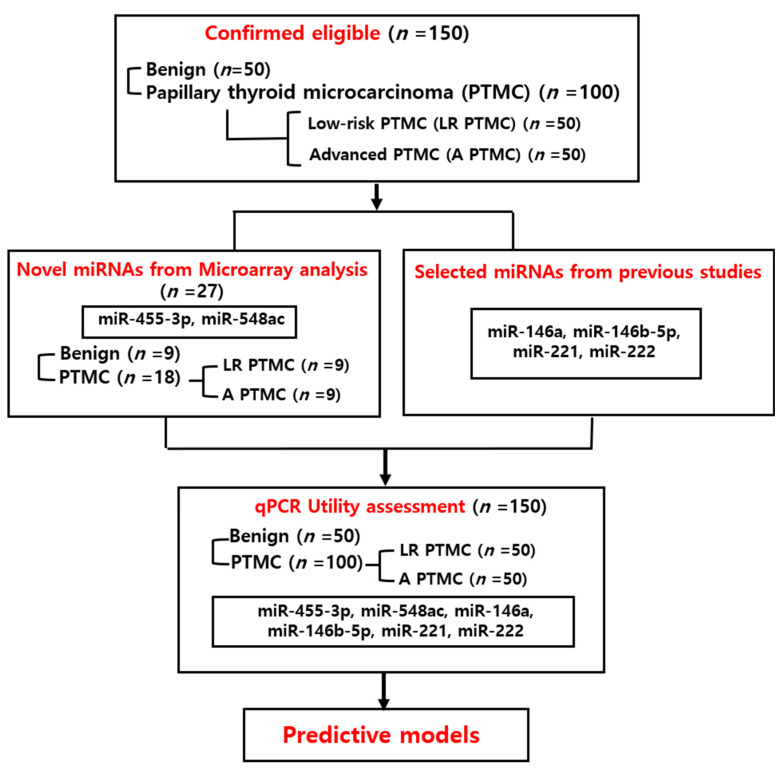
Summary of the study design.

For the discovery phase of candidate miRNA biomarkers, a subset of 27 patients (9 from each group) was randomly selected from the 150-patient study cohort for microarray analysis. Selection was stratified to maintain balance in age, sex, and tumor characteristics. This analysis served as an initial screening step, followed by validation of differentially expressed miRNAs in the entire cohort using TaqmanqPCR assay (Figure 1). Blood samples were collected from patients who provided written informed consent before surgery and no hemolyzed samples were included in the analysis.

### 2.3. Selection of miRNAs

Nine patients from each group were selected for microarray analysis of circulating plasma miRNAs (27 samples, including 9 with benign nodules and 18 with PTMC), based on frequency matching of factors such as age, sex, tumor size, and multifocality to identify novel miRNAs that are significant in PTMC (Table 2). Based on the microarray result, two miRNAs, miR-455-3p and miR-548ac, were selected as novel candidates for validation due to their significant differential expression. Additionally, miR-146a, miR-146b-5p, miR-221, and miR-222 were selected for research based on previous studies because they are known to be significant for PTC diagnosis, with expression levels increasing with greater aggressiveness [14,15,16,20,21]. Additionally, there is some research on circulating miRNAs related to these miRNAs [17,18].

### 2.4. Microarray

The RNA purity and integrity were assessed using an ND-2000 Spectrophotometer (NanoDrop, Wilmington, DE, USA) and an Agilent 2100 Bioanalyzer (Agilent Technologies, Palo Alto, CA, USA). The Affymetrix Genechip miRNA 4.0 array procedure (Affymetrix, Santa Clara, CA, USA) was performed following the manufacturer’s instructions. RNA samples (1000 ng) were labeled using the FlashTag Biotin RNA Labeling Kit (Genisphere, Hatfield, PA, USA). The labeled RNA was quantified, fractionated, and hybridized to the miRNA microarray according to standard procedures provided by the manufacturer. The labeled RNA was heated at 99 °C for 5 min, followed by 45 °C for 5 min. Subsequently, RNAarray hybridization was performed with agitation at 60 rotations per minute for 16 h at 48 °C using an Affymetrix 450 Fluidics Station. The chips were washed and stained using a GeneChip Fluidics Station 450 (Affymetrix). Chips were scanned using an Affymetrix GCS 3000 scanner. Signal values were determined using the Affymetrix GeneChip Command Console software (4.0) (Santa Clara, CA 95051, USA).

### 2.5. RNA Isolation

For plasma samples, the RNA was extracted using mirVana PARIS (Thermo Fisher Scientific, Waltham, MA, USA) according to the manufacturer’s protocol. Briefly, to isolate miRNA, 800 µL of plasma was mixed with an equal volume of 2× denaturing solution at 16 °C, and the mixture was incubated on ice for 5 min. Subsequently, 650 µL of acid-phenol was added, and the mixture was vigorously vortexed for 10 s. After centrifugation at 10,000× *g* for 10 min at 4 °C, the supernatant was transferred to a new tube, and 1.25 volumes of ethanol were added. Following a 5 min incubation at 16 °C, 600 µL of the mixture was loaded onto a filter cartridge placed in a collection tube and centrifuged at 10,000× *g* for 30 s at 4 °C. The filter cartridge was then washed with 600 µL of wash solution 1, followed by centrifugation at 10,000× *g* for 30 s at 4 °C. Next, the cartridge was washed twice with 500 µL of wash solution 2/3, each time centrifuged at 10,000× *g*, first for 30 s and then for 1 min at 4 °C. The filter cartridge was then transferred to a new collection tube, and total miRNA was eluted with 50 µL of elution solution, as per the manufacturer’s protocol, and stored at −80 °C following a final centrifugation at 10,000× *g* for 1 min.

### 2.6. Reverse Transcriptase Reactions

Reverse transcription (RT) was performed using the TaqMan miRNA RT kit (Applied Biosystems, Foster City, CA, USA). The reaction volume was set to 15 µL and included 5 µL of RNA extract, 1.5 µL of 10× RT buffer, 0.2 µL of RNase inhibitor (20 U/µL), 1 µL of reverse transcriptase (50 U/µL), 0.15 µL of 100 mM deoxyribonucleotide triphosphates, 3 µL of specific 5X miRNA primers (targeting miR-455-3p, miR-548ac, miR-146a, miR-146b-5p-5p, miR-221, and miR-222), and 4.15 µL of nuclease-free water. All miRNA primer sequences were purchased and used as validated products from Thermo Fisher Scientific. For complementary DNA (cDNA) synthesis, the reaction mixtures were incubated at 16 °C for 30 min, followed by 42 °C for 30 min, and a final step at 85 °C for 5 min.

### 2.7. Quantitative Real-Time PCR

Following reverse transcription, RT-qPCR was carried out using the Applied Biosystems 7900HT Sequence Detection System (Applied Biosystems, Waltham, MA, USA) and the TaqMan^®^ Universal PCR Master Mix (Thermo Fisher Scientific). Each 20 µL RT-qPCR reaction mixture contained 2 µL of cDNA, 10 µL of TaqMan^®^ Universal PCR Master Mix, 1 µL of specific 20XmiRNA primer (targeting miR-455-3p, miR-548ac, miR-146a, miR-146b-5p, miR-221, and miR-222), and 7 µL of nuclease-free water. The thermal cycling conditions were as follows: an initial incubation at 95 °C for 10 min, followed by 40 cycles of 95 °C for 15 s and 60 °C for 1 min. All the miRNA primer sequences (miR-455-3p, miR-548ac, miR-146a, miR-146b-5p, miR-221, and miR-222) were purchased from Thermo Fisher Scientific. Normalization was performed by using the differences between the cycle thresholds (delta CT) and the miRNA-191 expression levels to compute the delta CT/target gene delta CT ratio.

### 2.8. Statistics Analysis

Statistical analysis of fold change was performed using GraphPad Prism software, version 10 (GraphPad Software, La Jolla, CA, USA). Unless otherwise stated, all quantitative data were reported as the mean standard deviation from all samples. Wilcoxon rank sum tests were used to determine significant differences between groups. Receiver operating characteristic (ROC) curves were used to determine the diagnostic efficacy of the miRNAs. R version 4.3.1 (R Core Team, Vienna, Austria) was used for analysis. The area under the curve (AUC) indicated the discriminative ability of the dependent variable. The AUC results were interpreted as follows: 0.5–0.6, failed; 0.6–0.7, poor; 0.7–0.8, normal; 0.8–0.9, favorable; and 0.9–1, excellent discriminative ability. The Youden index was used to determine the best cutoff point that maximized both sensitivity and specificity. Statistical significance was set at *p* < 0.05.

## 3. Results

### 3.1. Comparison of Benign and PTMC

#### 3.1.1. Microarray Analysis Performed on the Benign Nodules and PTMC Groups

**Figure 2:**
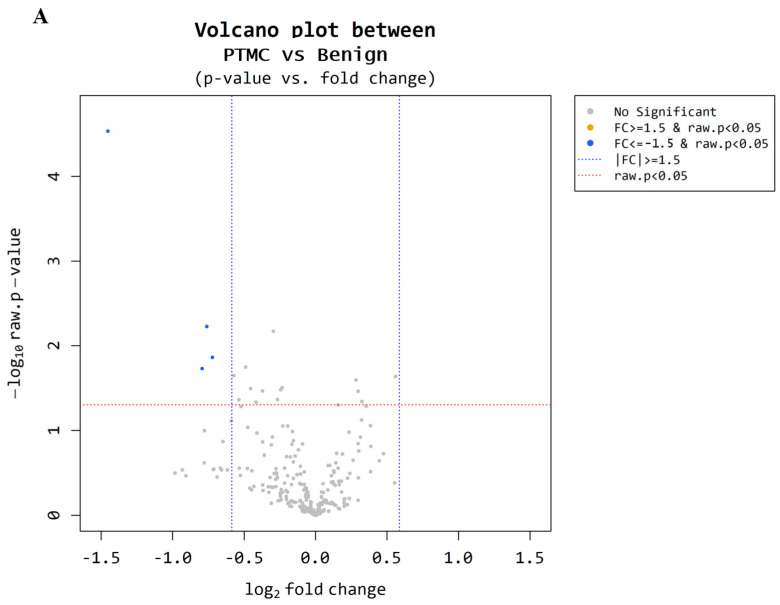

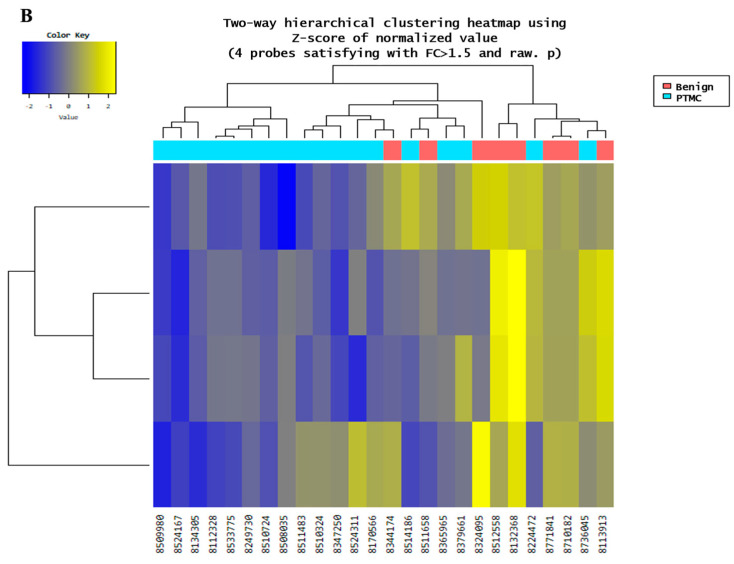
Microarray results for benign and PTMC. (**A**) A volcano plot illustrates differential expression of miRNAs between the benign and PTMC groups. Each point represents a miRNA, with the *x*-axis indicating the log2(1.5) fold change and the *y*-axis representing the statistical significance or −log10 raw *p* value. Four miRNAs (represented by the blue dots) exhibit a significant down regulation (*p*-value < 0.05). (**B**) The heatmap illustrates the miRNA expression patterns in the plasma of 9 benign groups and PTMC groups. Each column represents an individual patient, while each row represents miRNA. The color scale indicates higher expression in yellow and lower expression in blue.

Microarray analysis was performed to examine the circulating miRNA expression patterns in the plasma of patients with benign thyroid nodules (n = 9) and PTMCs (n = 18). Microarray data were filtered using a volcano plot to identify differentially expressed miRNAs in both groups. The results indicated that four miRNAs exhibited a 0.5~1.5-log2 fold decrease in expression and had a raw *p*-value ≤ 0.05 (Figure 2A). Through hierarchical clustering, differentially expressed miRNAs were identified and a heatmap was created to visualize the results (Figure 2B). However, two of these were excluded because they were unnamed sequences, i.e., ENSG00000239080 (Probe ID: 20533711), ENSG00000239080 (Probe ID: 20533712), as shown in Table 3. The expression of miR-455-3p decreased 2.73-fold (*p* < 0.001) in the PTMC group compared with that in the benign group, whereas the expression of miR-548ac decreased 1.69-fold (*p* < 0.05).

#### 3.1.2. Plasma miRNA TaqMan Assay Performed on the Benign Nodules and PTMC Groups

**Figure 3:**
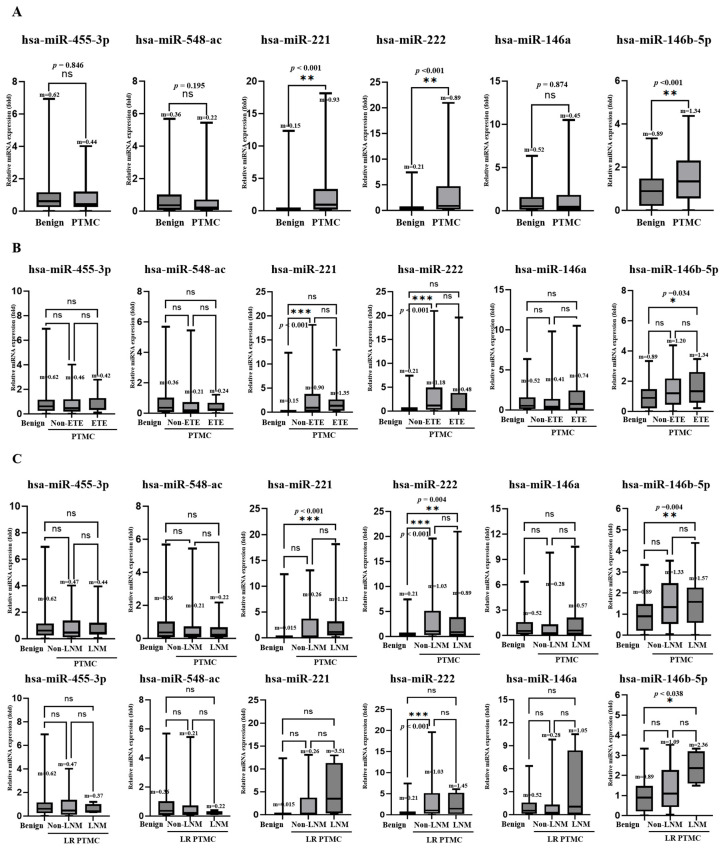
Real-time qPCR assay results for benign and PTMC. (**A**) Differences in miRNA expression between benign and PTMC. The graph shows the validation of plasma levels of 6 miRNAs in 50 patients with benign nodules and 100 patients with PTMC. Expression levels of miR-221, miR-222, and miR-146b were significantly upregulated in the PTMC group. (**B**) Differences in miRNA expression according to extrathyroidal extension. Circulating miRNA expression was analyzed in 50 individuals with benign nodules, 72 PTMC patients without extrathyroidal extension (non-ETE), and 28 PTMC patients with ETE. Although miR-146b-5p levels were higher in the ETE group compared to patients with benign nodules, no miRNA changes associated with ETE were observed in PTMC. (**C**) Differences in miRNA expression according to lymph node metastasis. Comparative analysis of plasma miRNA was conducted in 50 patients with benign nodules, 46 PTMC patients without lymph node metastasis (non-LNM), and 54 PTMC patients with LNM. Results indicated that miR-221, miR-222, and miR-146b-5p levels were significantly elevated in patients with LNM. However, no miRNAs changes associated with LNM were observed in PTMC (upper panel). Forty-six LR-PTMC patients without lymph node metastasis (non-LNM) and four LR-PTMC patients with LNM were analyzed. miR-146b-5p levels were significantly increased in LNM patients (lower panel). Statistical significance at * *p* < 0.05 ** *p* < 0.01 and *** *p* < 0.001.

A total of 150 patients (benign nodules = 50, low-risk PTMC = 50, and advanced PTMC = 50) were included. The expression levels of six miRNAs (miR-455-3p, miR-548ac, miR-221, miR-222, miR-146a, and miR-146b-5p) were analyzed by TaqMan RT-PCR. The results revealed that plasma levels of miR-221, miR-222, and miR-146b-5p were notably higher in patients with PTMC (*p* < 0.01, respectively) than in those with benign nodules. Compared with the benign nodule group, the fold changes of miR-221, miR-222, and miR-146b-5p were 2.67 ± 3.94, 2.87 ± 4.24, and 1.51 ± 1.10, respectively (Figure 3A). miR-146b-5p was increased in the ETE compared to the benign nodule group (Figure 3B). miR-221, miR-222, and miR-146b-5p were increased in the LNM with PTMC group compared to the benign nodule group, and miR-146b-5p was increased in the LNM with LR-PTMC group compared to the benign nodule group (Figure 3C). However, no miRNA changes associated with ETE and LNM were observed in either PTMC or LR-PTMC (Figure 3B,C).

#### 3.1.3. Differentiation Between Benign Nodules and PTMC Groups by ROC Curve

**Figure 4:**
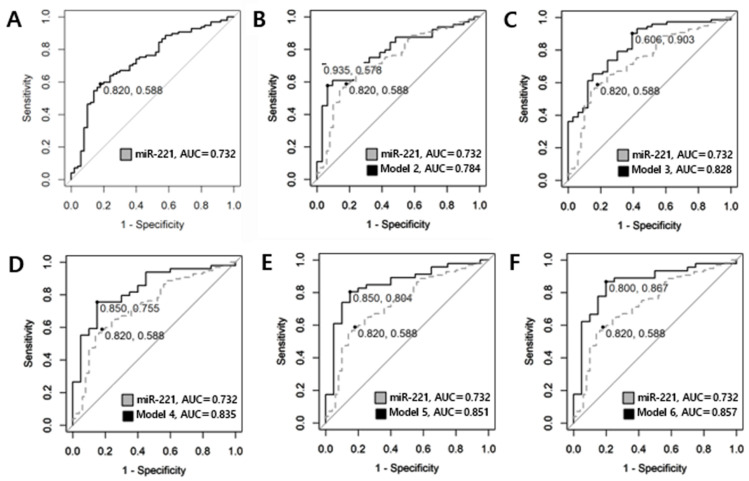
ROC curve utilized in comparing benign and PTMC. (**A**) single miRNA-221 represents a sensitivity of 0.588 and specificity of 0.820 with an AUC of 0.732. (**B**) The combination of miRNA-455-3p and miRNA-221 is illustrated, which represents a sensitivity of 0.578 and specificity of 0.935, and has the highest diagnostic value among Model 2, with an AUC of 0.784. (**C**) The combination of miRNA-548ac, miRNA-221, and miRNA-222 exhibits an AUC of 0.828, with a sensitivity of 0.903 and a specificity of 0.606. (**D**) The combination of miRNA-455-3p, miRNA-548ac, miRNA-221, and miRNA-222 is represented with an AUC of 0.835, a sensitivity of 0.755 and a specificity of 0.850. (**E**) The combination of miRNA-455-3p, miRNA-548ac, miRNA-146a, miRNA-221, and miRNA-222 has an AUC of 0.851, a sensitivity of 0.804 and a specificity of 0.850. (**F**) Model 6 illustrates the results for the combination of all six miRNAs (miR-455-3p, miR-548ac, miR-146a, miR-146b, miR-221, miR-222). The AUC increased to 0.857, indicating good diagnostic value, with a sensitivity of 0.867 and a specificity of 0.800.

ROC curve analysis was conducted to assess the predictive value of circulating plasma miRNAs for preoperative diagnosis in benign nodules and PTMC. Logarithmic transformation was employed to approximate the data distribution to a normal distribution. The AUCs of miR-455-3p, miR 548ac, miR-146a, miR-146b, miR-222 were 0.512 (95% CI, 0.386–0.638, *p* = 0.579), 0.578 (95% CI, 0.458–0.697, *p* = 0.903), 0.508 (95% CI, 0.410–0.606, *p* = 0.564), 0.640 (95% CI, 0.547–0.734, *p* = 0.003), and 0.703 (95% CI, 0.612–0.793, *p* < 0.001), respectively. Among single miRNAs, miR-221 exhibited the highest AUC of 0.732 (95% CI, 0.646–0.819, *p*-value < 0.001). With a cut-off value of −0.424, the sensitivity was determined to be 0.588, and the specificity was 0.820. Although the AUC exceeded 0.7, suggesting a moderate level of discriminative value, the sensitivity was observed to be relatively low. To enhance diagnostic accuracy of circulating plasma miRNAs, a model was constructed by combining two or more miRNAs and generating a ROC curve to evaluate their performance. The model numbers represent the number of miRNAs combined. Table 4 and Figure 4 display the results for single miRNAs as well as the best combination selected for each model. The concept of Bayesian Information Criterion (BIC) was used to select the most suitable model from various combinations. A model with increased AUC while maintaining a lower BIC was selected. The model incorporating all six miRNAs yielded the highest AUC at 0.857 (95% CI, 0.753–0.960, *p* < 0.001) while also exhibiting a relatively low BIC (Figure 3). Sensitivity increased to 0.867, and the specificity was 0.800. The calculation formula of the model is Logit(P) = 1.383 − 0.06 × (log-scaled value of miRNA-455-3p) − 0.214 × (log-scaled value of miRNA-548ac) − 0.207 × (log-scaled value of miRNA-146a) − 0.114 × (log-scaled value of miRNA-146b) + 0.544 × (log-scaled value of miRNA-221) + 0.397 × (log-scaled value of miRNA-222). By inputting the expression values of each miRNA into this formula, the probability of diagnosing PTMC can be obtained through the odds value.

### 3.2. Low-Risk PTMC and Advanced PTMC

#### 3.2.1. Microarrays Performed on the Low-Risk and Advanced PTMC Groups

**Figure 5:**
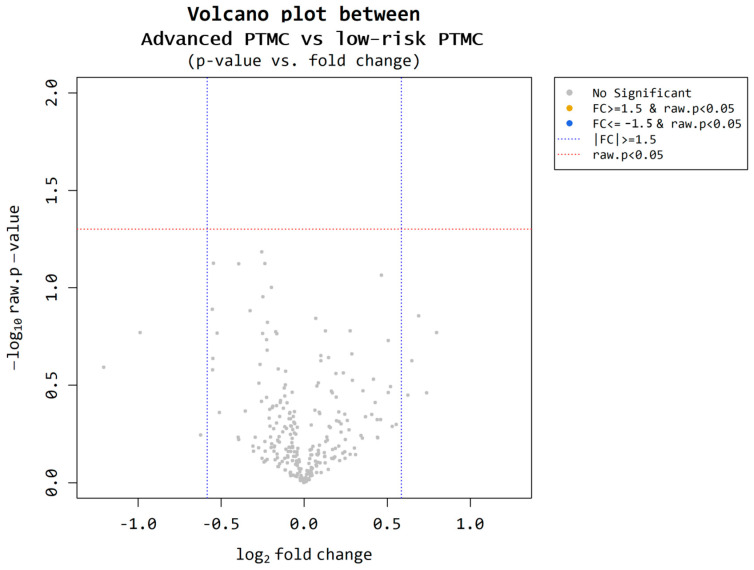
Microarray results for low-risk PTMC and advanced PTMC. A volcano plot illustrates differential expression of miRNAs between the low-risk PTMC group and advanced PTMC group. Each point represents a miRNA, with the *x*-axis indicating the log2(1.5) fold change and the *y*-axis representing the statistical significance or −log10 raw p value. There is no significant upregulation, with a fold change greater than 1.5 and statistical significance (*p*-value < 0.05).

Nine participants were selected from each cohort (low-risk PTMC = 50; advanced PTMC = 50). Based on the microarray results, we compared the two groups. Utilizing volcano plot analysis, miRNAs meeting the criteria of a fold change of ≥1.5 and *p* < 0.05 were identified (Figure 5). However, no miRNAs satisfying these criteria were identified.

#### 3.2.2. Plasma miRNA TaqMan Assay Performed on the Low-Risk and Advanced PTMC Groups

**Figure 6:**
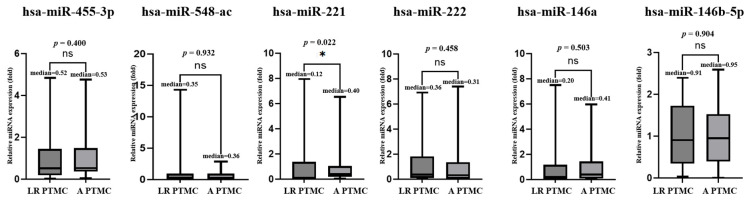
Real-time qPCR results for low-risk (LR)and advanced(A) PTMC. The graph shows the validation of plasma levels of 6 miRNAs in 50 patients with low-risk PTMC and 50 patients with advanced PTMC. miR-221 showed significant downregulation.* Indicates the statistical at the *p* < 0.05, ns: not significant.

In the microarray analysis, no novel miRNA candidates were identified to distinguish between the low- and advanced-risk groups. Therefore, we included miR-455-3p and miR-548ac, which were significant in the microarray analysis, to distinguish between benign nodules and PTMC. The expression levels of all six miRNAs in low-risk (n = 50) and advanced PTMC (n = 50) were analyzed using TaqMan RT-PCR. The findings indicated that miR-221 showed significant downregulation in plasma miRNA expression levels. The results revealed that the *p*-values of plasma miR-455-3p, miR-548ac, miR-221, miR-222, miR-146a, and miR-146b-5p were 0.489, 0.428, 0.982, 0.922, 0.802, and 0.985, respectively, and the fold changes were 1.21 ± 1.30, 0.74 ± 0.87, 0.99 ± 1.43, 1.03 ± 1.67, 0.92 ± 1.24, and 1.00 ± 0.71, respectively (Figure 6).

#### 3.2.3. Differentiation Between Low-RISK and ADVANCED PTMC by ROC Curve

**Figure 7:**
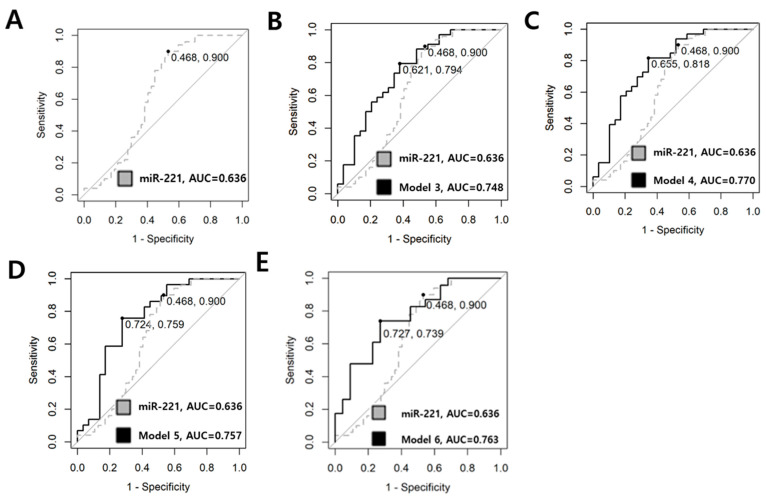
ROC curve utilized in comparing low-risk and advanced PTMC. (**A**) Dotted line represents the diagnostic value of the single miRNA-221. The sensitivity was 0.900, specificity was 0.468, and the AUC was 0.636. (**B**) The combination of miRNA-455.3p, miRNA-221, and miRNA-222 is illustrated. The AUC is 0.748, with a sensitivity of 0.794 and a specificity of 0.621. (**C**) The combination of miRNA-455-3p, miRNA-146b, miRNA-221, and miRNA-222 exhibits an AUC of 0.770, with a sensitivity of 0.818 and a specificity of 0.655. (**D**) The combination of miRNA.455-3p, miRNA-146a, miRNA-146b, miRNA-221, and miRNA-222 is represented with an AUC of 0.757, a sensitivity of 0.759 and a specificity of 0.724. (**E**) Model 6 illustrates the results for the combination of all six miRNAs (miR-455-3p, miR-548ac, miR-146a, miR-146b, miR-221, miR-222). The AUC increased to 0.763, indicating normal diagnostic value, with a sensitivity of 0.739 and a specificity of 0.727.

When distinguishing between low-risk and advanced PTMC, miR-221 exhibited the highest AUC of 0.636 (95% CI 0.518–0.754, *p* < 0.001); however, the diagnostic value was poor, with a sensitivity of 0.900 and specificity of 0.468. WE investigated whether combining two or more miRNAs could enhance the diagnostic value. Table 5 displays the results for single miRNAs and the best diagnostic values for each model number, and Figure 7 depicts the ROC curves of combinations where diagnostic values were enhanced above the normal level. In Model 3, the combination of miRNA-455-3p, miRNA-221, and miRNA-222 yielded an AUC of 0.748 (95% CI, 0.624–0.873, *p* < 0.001). In Model 4, the combination of miRNA-455.3p, miRNA-146b-5p, miRNA-221, and miRNA-222 resulted in an AUC of 0.770 (95% CI, 0.649–0.886, *p* < 0.001). In Model 5, the combination of miRNA.455-3p, miRNA-146a, miRNA-146b-5p, miRNA-221, and miRNA-222 exhibited an AUC of 0.757 (95% CI 0.629–0.886, *p* < 0.001). The model incorporating all six miRNAs yielded an increased AUC of 0.763 (95% CI, 0.623–0.903, *p* = 0.001) with a relatively low BIC, representing the best model. Specificity and sensitivity increased to 0.727 and 0.739, respectively. The calculation formula of the best model is Logit(P) = 0.829 + 0.361 × (log-scaled value of miRNA-455-3p) − 0.029 × (log-scaled value of miRNA-548ac) + 0.239 × (log-scaled value of miRNA-146a) − 0.275 × (log-scaled value of miRNA-146b) + 0.417 × (log-scaled value of miRNA-221) − 0.53 × (log-scaled value of miRNA-222).

## 4. Discussion

Ultrasound and fine-needle aspiration are the most basic tests for evaluating thyroid cancer. FNA is performed on suspicious thyroid nodules detected on ultrasonography. The sensitivity of FNA ranges from 65% to 98%, while the specificity ranges from 72% to 100% [22]. In particular, precise needle placement can be challenging for nodules smaller than 1 cm. ATA guideline recommends FNA for suspicious malignant thyroid nodules larger than 1 cm on ultrasound [18] and the Korean Thyroid Association (KTA) selectively performs FNA on suspicious malignant nodules smaller than 1 cm depending on location [23]. Even though uncommon, some complications such as vascular injury (0–1.9%), hematoma, and hemoptysis can occur after FNA [23]. Therefore, a simple and safe method based on blood sampling, such as the detection of plasma-derived miRNAs or circulating cancer cells, may be helpful in the early detection of PTMC.

Recently, active research has been focused on the application of molecular tests in clinical assessment. It is accepted that miRNAs are stable enough to be used in clinical specimens such as plasma or serum, providing high specificity, sensitivity and accessibility [24]. Therefore, many articles focus on the development of diagnostic methods utilizing circulating miRNAs, such as serum, plasma, blood cells, and microvesicles, which enable minimally invasive testing [25]. For instance, the diagnosis of colorectal cancer can be achieved with a sensitivity and specificity of 90% through the measurement of plasma levels of miRNA-21-5p [26].

In hepatocellular carcinoma, miRNA-122-5p from serum or plasma samples could aid in distinguishing hepatocellular carcinoma patients with approximately 82% sensitivity and 83% specificity [27]. Besides the miRNAs mentioned above, extensive research is being conducted on circulating miRNAs, covering various cancers including lung, colorectal, breast, and prostate cancer [28,29], as well as non-cancerous diseases such as heart failure [30], and diabetes [31].

Research on miRNA expression in PTC has also been conducted, with numerous studies suggesting that miRNAs extracted from tumor tissue, plasma, and serum are useful in the diagnosis and prognostication of PTC [14,16,17,18,19,32,33].

Kondrotienė et al. [34] performed a study using quantitative RT-PCR on various tissue-derived miRNAs to distinguish between the PTC and control groups, finding that miR-146, miR-221, and miR-222 had AUCs of 0.770, 0.729, and 0.720, respectively. Lee et al. [17] found that plasma-derived miR-146b and miR-155 can distinguish between PTC and benign nodules, suggesting that they could serve as valuable serological markers for PTC. Liu et al. [18] performed a meta-analysis on the diagnostic value of serum or plasma miRNAs for PTC and found that miR-222 and miR-146b have high specificity for the diagnosis of PTC. However, in a systematic review, Geropoulos et al. [35] found that the expression levels of some circulating miRNAs, including miR-146b, miR-221, and miR-222, are associated with thyroid tumor size.

Our study contributes to research in the field by investigating whether plasma miRNAs can effectively distinguish between benign nodules and PTCs smaller than 1 cm. Combining multiple miRNAs proved more appropriate for distinguishing benign nodules from PTMC. Qiao et al. [36] confirmed that although the AUC of a single miRNA may be unsatisfactory, combining two or more miRNAs for diagnosis can yield improved diagnostic value. In our study, we explored all possible combinations and constructed ROC curves. Ultimately, the combination of six miRNAs (miR-455-3p, miR-548ac, miR-221, miR-222, miR-146a, and miR-146b-5p) achieved superior diagnostic value with an AUC of 0.857. This indicates strong diagnostic performance with high sensitivity (86.7%) and specificity (80.0%) for differentiating between cases of benign nodules and PTMC. The result aligns with previously reported values for tissue-based miRNA markers (e.g., 0.720–0.770 for miR-146b, miR-221, and miR-222) [17,35]. Afirma and ThyroSeq, two widely used commercial molecular tests, have reported AUCs of approximately 0.78 and 0.87, respectively, in studies involving indeterminate thyroid nodules [37,38]. While these are tissue-based and not specifically designed for PTMC, their reported AUCs could provide a useful reference point. Our plasma-derived six-miRNA panel demonstrated a comparable diagnostic performance with an AUC of 0.857, suggesting that circulating miRNA panels could offer comparable diagnostic utility in a less invasive format.

Surgery is the primary treatment for PTMCs. Since most cases are low-risk, lobectomy is generally sufficient. However, in some cases, completion thyroidectomy is required because multiple central metastases or vascular invasions are discovered after lobectomy. Alternatively, aggressive PTMC may be accompanied by lateral cervical metastasis or vocal cord paralysis. Some patients initially undergo hemithyroidectomy as a low-risk case, but are later found to have intermediate- or high-risk factors after surgery, necessitating additional surgery or RAI. In current clinical practice, it is difficult to accurately distinguish between low-risk and advanced PTMC before surgery. The development of a predictive method for aggressiveness before surgery could alleviate patient discomfort and reduce the associated social costs by minimizing the need for additional surgical interventions.

In terms of clinical applicability, cost-effectiveness must be carefully considered. In this study, the cost of analyzing the proposed six-miRNA plasma panel was approximately USD 250 per patient, excluding indirect laboratory expenses such as personnel time, consumables, and equipment usage. Although a comprehensive health economic evaluation was not performed, this estimated cost is substantially lower than that of commercial tissue-based or NGS-based molecular assays, such as ThyroSeq or Afirma. These findings suggest that plasma-based miRNA testing may offer a relatively affordable and accessible option for future clinical applications.

Several systematic reviews have revealed that circulating miRNAs, specifically miR-146, miR-221, and miR-222, are upregulated in patients with PTC with ETE or lymph node metastasis [20,34]. They are known to be regulated by redox-sensitive transcription factors like NF-κB [39]. Oxidative stress is increasingly recognized as a contributor to tumor progression and metastatic potential. Therefore, redox-mediated regulation of these miRNAs may provide a biological explanation for their association with aggressive features in PTC.

Our study investigated whether this discriminatory capability extends to PTMC. Zhang et al. discovered that circulating levels of miR-222 and miR-21 were significantly elevated in patients with PTMC with ETE and metastatic lymph nodes, although their study included only 30 patients [40]. Beyond simple fold-change comparisons, our study aimed to provide practical assistance in selecting surgical approaches for clinical use through ROC curve analysis with a larger study population of 100 participants. However, when analyzing the expression of single miRNAs alone, no statistically significant differences were observed between the low-risk and advanced-stage groups. Nonetheless, the combination of plasma miRNAs (miR-455-3p, miR-548ac, miR-221, miR-222, miR-146a, and miR-146b-5p) resulted in moderate diagnostic efficacy, with an increase in AUC from 0.636 to 0.763, compared with using a single miRNA. The sensitivity improved to 73.9% and the specificity to 72.7%. Although this study achieved discriminatory value only up to the normal level, the outcome demonstrated the potential for a high diagnostic value when combining selected miRNAs appropriately. If this panel is clinically validated and implemented, it could be integrated into active surveillance protocols to enable simple, serial risk assessments that stratify patients into low risk and aggressive groups. Low-risk individuals might safely undergo prolonged surveillance, and aggressive patients could undergo earlier surgical intervention. This panel also could help determine the optimal surgical extent and reduce the need for repeat surgeries.

This study had some limitations. It was limited to a single center in Korea. Furthermore, plasma miRNAs are not exclusively produced in cancerous tissues and can vary depending on inflammation or other systemic conditions. These factors could potentially lower the AUC for clinical use. Although several key miRNAs (miR-221, miR-222, miR-146a, and miR-146b) have been consistently reported as overexpressed in PTC tissues, supporting their tumor association [32,41], novel candidates identified by microarray (miR-455-3p and miR-548ac) need further validation using matched tumor samples. Alternatively, future studies could consider using exosome-derived miRNAs, as these vesicles are actively secreted by tumor cells and may more accurately reflect the molecular profile of thyroid cancer. Focusing on exosomal miRNAs could improve tumor specificity and help reduce background signals from non-cancerous sources [42,43].

The absence of an independent validation cohort also represents a limitation of the study. Although we attempted external validation using publicly available datasets such as TCGA, these resources lack the necessary clinical annotations, particularly tumor size, to distinguish microcarcinomas, which is essential for PTMC-specific analysis.

Multiple-testing correction was also not applied in the microarray analysis due to the limited sample size in the discovery cohort. Further studies with larger sample sizes should incorporate appropriate correction methods, such as false discovery rate adjustment, to improve statistical reliability. Moreover, the relatively wide confidence intervals observed in the ROC analyses also suggest that further studies are needed to confirm the reproducibility and precision of the findings. Therefore, validation using a larger and independent patient cohort is necessary for practical clinical application.

In future large-scale validation studies, it would be beneficial to include angioinvasion as a prognostic variable. Unfortunately, this parameter was not assessed in the present study due to the limited number of cases with confirmed vascular invasion on histopathological examination. However, vascular invasion is a well-established and critical prognostic factor in thyroid carcinoma. Moreover, there is currently no reliable preoperative method that can predict vascular invasion. If circulating miRNAs can help predict this feature preoperatively, it would represent a significant advancement in risk stratification and surgical decision-making.

## 5. Conclusions

In conclusion, this study investigated whether the expression levels of miRNAs in plasma collected from patients with PTMC could be used for diagnosis and prognosis prediction. The combination of six miRNAs (miR-455-3p, miR-548ac, miR-221, miR-222, miR-146a, and miR-146b-5p) has the potential to achieve high diagnostic value and a satisfactory level of prognostic discrimination.

## Figures and Tables

**Table 1:** Clinical characteristics of 150 enrolled patients.

Characteristics	Benign (n = 50)	Low-Risk PTMC (n = 50)	Advanced PTMC (n = 50)
Gender			
Male	13	13	13
Female	37	37	37
Age (yr, mean ± SD)	48.56 (±12.34)	48.51 (±11.53)	48.74 (±11.70)
Tumor size (cm ± SD)	2.38 (±1.34)	0.66 (±0.17)	0.67 (±0.18)
Multifocality, n (%)			
Yes	-	22 (44)	22 (44)
No	-	28 (56)	28 (56)
ETE, n(%)			
No	-	42(84)	31(62)
Minimal	-	8(16)	18(36)
Gross		0 (0)	1(2)
CLNM, n (%)			
Yes	-	4 (8)	47 (94)
No	-	46 (92)	3 (6)
LLNM, n (%)			
Yes	-	0 (0)	20 (40)
No	-	50 (100)	30 (60)
BRAF mutation, n (%)			
Not tested	-	40 (80)	39 (78)
Positive	-	1 (2)	7 (14)
Negative	-	9 (18)	4 (8)
Number of LN metastasis cases Average n (min,max)	0 (0,0)	0.04 (0,1)	8.42 (5,20)
Surgery, n (%)			
HT	31 (62)	2 (4)	0 (0)
HT with CCND **	11 (22)	16 (32)	5 (10)
TT	3 (6)	0 (0)	0 (0)
TT with CCND **	5 (10)	32 (64)	25 (50)
TT with CCND, LND	0 (0)	0 (0)	20 (40)

** Central compartment neck dissection (CCND) was performed for prophylactic and therapeutic purposes. ETE: Extrathyroidal extension; CLNM: central lymphnode metastasis; LLNM: lateral lymph node metastasis; HT: hemithyroidectomy; LN: lymphnode; TT: total thyroidectomy; CCND: central compartment neck dissection; LND: lateral neck dissection.

**Table 2:** Clinical characteristics of 27 microarray subjects.

Characteristics	Benign (n = 9)	Low-Risk PTMC (n = 9)	Advanced PTMC (n = 9)
Gender			
Male	2	2	2
Female	7	7	7
Age (years, mean ± SD)	50.89 (±10.71)	49.22 (±10.70)	47.78 (±10.49)
Tumor size (cm ± SD)	2.84 (±1.60)	0.63 (±0.19)	0.7 (±0.16)
Multifocality, n(%)			
Yes	-	2	2
No	-	7	7

**Table 3:** Identification of plasma-derived miRNA that differentiate benign and PTMC.

miRNA Base	PTMC/Benign Fold Change	PTMC/Benign Raw *p*-Value
has-miR-455-3p	−2.736502	0.00002907
has-miR-548ac	−1.694879	0.00591083
-	−1.734454	0.01860101
-	−1.648868	0.01374299

has-miR: Homo sapiens microRNA, indicating microRNA found in humans.

**Table 4:** Diagnostic value of miRNAs in distinguishing between benign and PTMC.

Model	miRNA	AUC (95% CI)	Sensitivity (95% CI)	Specificity (95% CI)	Cut-Off Value (Log Transformed)	*p*-Value	BIC
1	miRNA-455-3p	0.512 (0.386, 0.638)	0.522 (0.418, 0.642)	0.613 (0.419, 0.774)	−0.774	0.579	131.5
1	miRNA-548ac	0.578 (0.458, 0.697)	0.533 (0.427, 0.640)	0.647 (0.500, 0.794)	−1.465	0.903	142.8
1	miRNA-146a	0.508 (0.410, 0.606)	0.387 (0.290, 0.484)	0.735 (0.592, 0.857)	−1.648	0.564	192.8
1	miRNA-146b	0.640 (0.547, 0.734)	0.480 (0.378, 0.582)	0.780 (0.660, 0.880)	0.419	0.003	192.3
1	miRNA-221	0.732 (0.646, 0.819)	0.588 (0.485, 0.691)	0.820 (0.720, 0.920)	−0.424	<0.001	176.8
1	miRNA-222	0.703 (0.612, 0.793)	0.626 (0.525, 0.717)	0.714 (0.571, 0.837)	−0.852	<0.001	179.8
2	miRNA-455-3p + miRNA-221	0.784 (0.690, 0.879)	0.578 (0.469, 0.703)	0.935 (0.839, 1.000)	-	<0.001	112.6
3	miRNA-548ac + miRNA-221 + miRNA-222	0.828 (0.745, 0.911)	0.903 (0.833, 0.958)	0.606 (0.424, 0.758)	-	<0.001	115.5
4	miRNA-455-3p + miRNA-548ac + miRNA-221 + miRNA-222	0.835 (0.732, 0.938)	0.755 (0.633, 0.857)	0.850 (0.700, 1.000)	-	<0.001	93.7
5	miRNA-455-3p + miRNA-548ac + miRNA-146a + miRNA-221 + miRNA-222	0.851 (0.748, 0.954)	0.804 (0.674, 0.913)	0.850 (0.650, 1.000)	-	<0.001	90.1
6	miRNA-455-3p + miRNA-548ac + miRNA-146a + miRNA-146b +miRNA-221 + miRNA-222	0.857 (0.753, 0.960)	0.867 (0.756, 0.956)	0.800 (0.600, 0.950)	-	<0.001	88.6

The model number indicates the quantity of miRNAs combined. From combinations of two or more, the best result from each combination is displayed. The *p*-values signify a significant difference from an AUC of 0.5. AUC: area under the receiver operating characteristic curve; BIC:Bayesian Information Criterion.

**Table 5:** Diagnostic value of miRNAs in distinguishing between low-risk and advanced PTMC.

Model	miRNA	AUC (95% CI)	Sensitivity (95% CI)	Specificity (95% CI)	Cut-Off Value (Log Transformed)	*p*-Value	BIC
1	miRNA-455.3p	0.560 (0.419, 0.701)	0.943 (0.857, 1.000)	0.250 (0.125, 0.406)	−1.804	0.203	99.3
1	miRNA-548ac	0.506 (0.373, 0.639)	0.400 (0.257, 0.571)	0.700 (0.550, 0.850)	−0.797	0.468	112.3
1	miRNA-146a	0.540 (0.421, 0.659)	0.614 (0.455, 0.750)	0.531 (0.388, 0.673)	−1.146	0.254	137.3
1	miRNA-146b	0.519 (0.403, 0.635)	0.521 (0.375, 0.667)	0.580 (0.460, 0.720)	0.433	0.373	144.9
1	miRNA-221	0.636 (0.518, 0.754)	0.900 (0.820, 0.980)	0.468 (0.319, 0.596)	−1.524	0.011	135.3
1	miRNA-222	0.543 (0.428, 0.658)	0.245 (0.122, 0.367)	0.880 (0.780, 0.960)	−2.284	0.772	145.7
2	miRNA.455-3p + miRNA-221	0.681 (0.540, 0.821)	0.971 (0.914, 1.000)	0.448 (0.276, 0.621)		0.006	92.8
3	miRNA-455-3p + miRNA-221 + mRNA-222	0.748 (0.624, 0.873)	0.794 (0.647, 0.912)	0.621 (0.448, 0.793)		<0.001	91.3
4	miRNA-455-3p + miRNA-146b + miRNA-221+ miRNA-222	0.770 (0.649, 0.891)	0.818 (0.667, 0.939)	0.655 (0.483, 0.828)		<0.001	91.7
5	miRNA-455-3p + miRNA-146a + miRNA-146b + miRNA-221 + miRNA-222	0.757 (0.629, 0.886)	0.759 (0.586, 0.897)	0.724 (0.552, 0.862)		<0.001	92.6
6	miRNA.455-3p + miRNA-548ac + miRNA-146a + miRNA-146b + miRNA-221 + miRNA-222	0.763 (0.623, 0.903)	0.739 (0.565, 0.913)	0.727 (0.545, 0.909)		0.001	77.8

The model number indicates the quantity of miRNAs combined. From combinations of two or more, the best result from each combination is displayed. The *p*-values signify a significant difference from an AUC of 0.5.

## Data Availability

The data supporting this study are not publicly available due to patient confidentiality.

## Abbreviations

The following abbreviations are used in this manuscript:

PTC	Papillary thyroid carcinoma
PTMC	Papillary thyroid microcarcinoma
US	Ultrasonography
LND	Lateral neck dissection
RAI	Radioactive iodine
miRNA	MicroRNA
ETE	Extrathyroidal extension
RT-PCR	Real-time polymerase chain reaction
AUC	Area under the curve
ROC	Receiver operating characteristic
TT	Total thyroidectomy
CCND	Central compartment neck dissection.
FNA	Fine needle aspiration
